# Amoxicillin-Triggered Rash in Latent Epstein-Barr Virus Infection: A Case of Kawasaki Disease Mimicry in a Seven-Year-Old Girl

**DOI:** 10.7759/cureus.77943

**Published:** 2025-01-24

**Authors:** Hirofumi Watanabe, Shigehiro Sainokami, Natsuho Adachi, Keiichi Takizawa, Shoichiro Kanda

**Affiliations:** 1 Pediatrics, The University of Tokyo, Tokyo, JPN

**Keywords:** aminobenzyl group, amoxicillin-associated rash, epstein-barr virus infections, infectious mononucleosis, kawasaki disease (kd)

## Abstract

Amoxicillin-associated rash is a well-documented phenomenon in Epstein-Barr virus (EBV)-related infectious mononucleosis. This case highlights a notable course where amoxicillin was administered during the latent phase of EBV infection, and the characteristic rash appeared following the clinical onset of infectious mononucleosis. A seven-year-old girl received amoxicillin for acute otitis media during EBV's latent phase. After developing fever, conjunctival injection, lip redness, cervical lymphadenopathy, and a generalized rash, she was initially diagnosed with Kawasaki disease and treated with intravenous immunoglobulin and aspirin. Serological testing later confirmed acute EBV infection, and the rash was attributed to prior amoxicillin use.

This case illustrates that aminopenicillins, such as amoxicillin, can trigger characteristic rashes after the onset of infectious mononucleosis, even when administered during the infection’s latent phase. It highlights the importance of detailed medication history-taking, particularly regarding drug use during the latent period, and the consideration of viral etiologies in febrile illnesses with rash, especially when symptoms overlap with systemic inflammatory conditions.

## Introduction

Infectious mononucleosis is a disease primarily caused by the initial infection with the Epstein-Barr virus (EBV) that establishes lifelong latency in B cells [1]. It is characterized by the classic triad of persistent fever, tonsillitis or pharyngitis, and cervical lymphadenopathy. Additional clinical features may include fatigue, hepatosplenomegaly, and atypical lymphocytosis, reflecting the systemic immune response to EBV infection. While the disease is typically self-limiting, complications such as airway obstruction, splenic rupture, and neurological manifestations can occur, necessitating careful clinical evaluation. It most commonly occurs in individuals in their teens and twenties, with a latent period of six weeks [2].

Amoxicillin rash is a well-recognized phenomenon associated with EBV infection, particularly in patients with infectious mononucleosis who are prescribed amoxicillin or other aminopenicillins. Amoxicillin rash is a non-allergic, immune-mediated reaction. Typically emerging 4 to 10 days after antibiotic initiation, it presents as a widespread, symmetric maculopapular eruption, often beginning on the trunk and spreading to the extremities with a tendency to coalesce into erythematous patches. Unlike allergic drug rashes, it is usually non-pruritic and not associated with anaphylaxis [3]. The rash resolves spontaneously within a week after discontinuation but may persist for up to two weeks with mild desquamation. Therefore, caution is required when prescribing amoxicillin to patients presenting with tonsillitis or pharyngitis.

Kawasaki disease is an acute febrile illness of childhood characterized by prolonged fever, bilateral nonexudative conjunctival injection, polymorphous rash, erythema and edema of the extremities, cervical lymphadenopathy, and mucosal inflammation, including cracked lips and a "strawberry tongue." Inflammatory changes in the coronary arteries can develop early in the disease course, making timely recognition essential for preventing complications.

Kawasaki disease and amoxicillin rash share several overlapping clinical features, including prolonged fever, exanthema, and cervical lymphadenopathy. While Kawasaki disease is diagnosed based on clinical criteria, infectious mononucleosis is confirmed through serological testing for EBV antibodies. Due to these differences in diagnostic approach, the two conditions are often identified at different time points, making differentiation challenging at the initial presentation.

Here, we report a case in which amoxicillin was prescribed prior to the onset of infectious mononucleosis and a rash developed after the onset of infectious mononucleosis.

## Case presentation

A seven-year-old girl presented with chief complaints of fever, conjunctival injection, lip redness, cervical lymphadenopathy, and rash. Her past medical history was unremarkable, and she had no significant prior health issues. Her family history revealed that her younger brother was diagnosed with Kawasaki disease during infancy.

The current illness began 14 days prior to her hospitalization when she developed a fever. She visited a local otolaryngology clinic and was diagnosed with acute otitis media. She was subsequently prescribed a five-day course of amoxicillin. Following the initial treatment, her fever promptly subsided, and she remained stable for a brief period. However, four days prior to hospitalization, she began experiencing symptoms of cough, sore throat, nasal discharge, and fever. She revisited a local clinic, where she was diagnosed with acute bronchitis and prescribed clarithromycin. Despite this, her fever persisted, and on the following day, a generalized rash associated with itching and pain appeared in the evening. The day after that, as the symptoms showed no improvement, she returned to the clinic. The rash was suspected to be drug-induced, leading to the discontinuation of clarithromycin. Nevertheless, her symptoms persisted, prompting another visit to the local clinic. This time, she was diagnosed with Kawasaki disease and referred to our hospital for further evaluation and management. A rapid antigen test for adenovirus conducted at the clinic returned negative.

**Figure 1:**
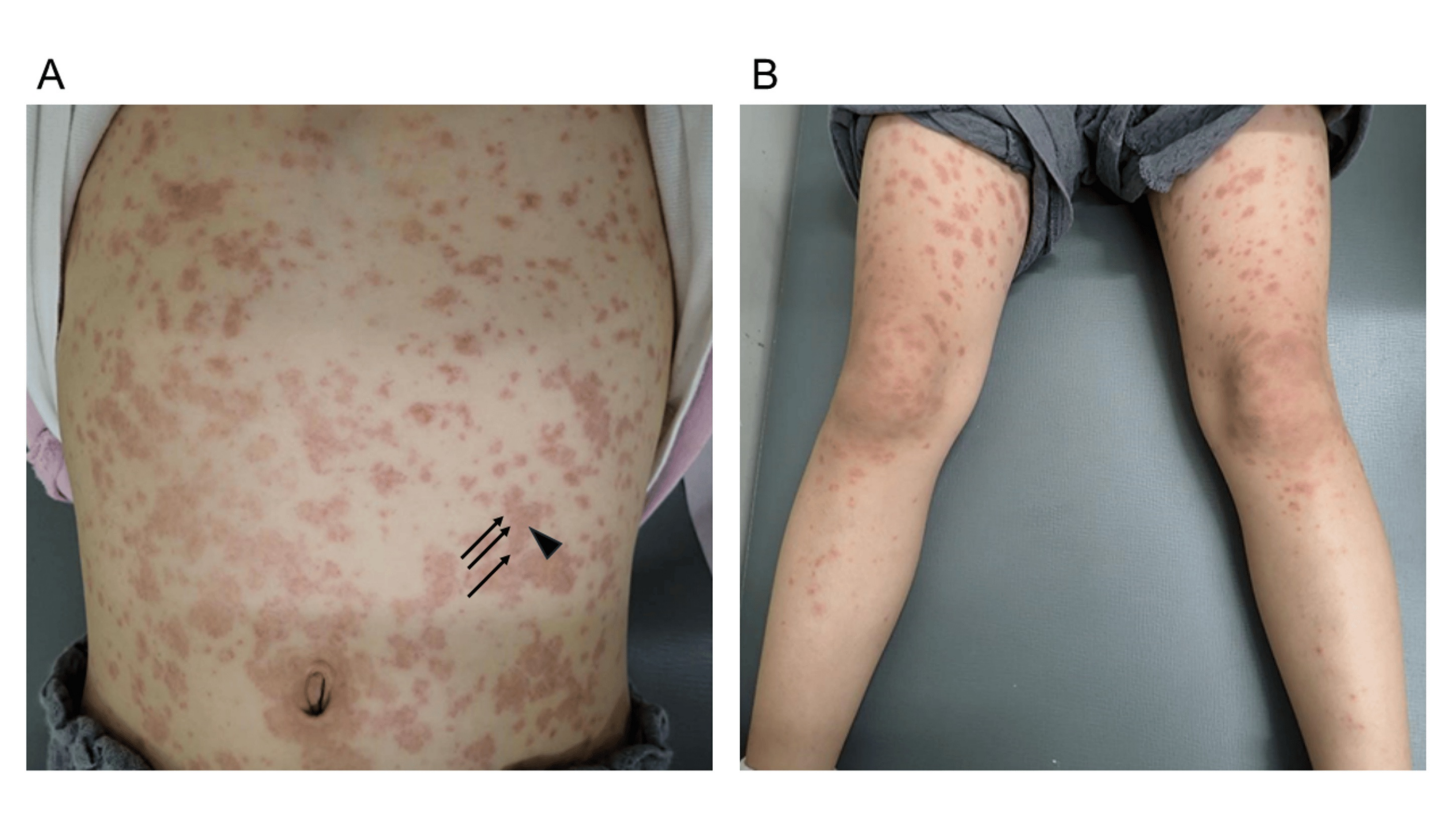
Rash observed in the patient Multiple scattered erythematous lesions measuring a few millimeters in diameter (arrows) were observed on the trunk and extremities, with surrounding purpuric macules up to fingertip size (arrowhead), exhibiting a tendency to coalesce. No significant mucosal involvement was noted.

Upon admission, her vital signs revealed a body temperature of 39.5°C, a heart rate of 130 beats per minute, blood pressure of 114/88 mmHg, and a SpO₂ level of 95% on room air. She appeared slightly lethargic on examination. Physical findings included conjunctival injection, lip redness, and white exudate on the tonsils. Tender bilateral cervical lymphadenopathy was noted. Multiple scattered erythematous lesions were observed on the trunk and extremities, some interspersed with purpuric macules up to fingertip size, exhibiting a tendency to coalesce and accompanied by itching and pain (Figure 1).

Cardiovascular examination revealed regular heart sounds with no murmurs, and breath sounds were clear upon auscultation. The abdominal examination showed no evidence of distension or increased bowel sounds. At the time of hospitalization, the venous blood gas analysis revealed no abnormalities. The platelet count was 187,000/μL. Although there was no increase in the white blood cell count, the C-reactive protein (CRP) level was elevated at 8.29 mg/dL. The serum sodium level was slightly decreased at 132 mmol/L. Blood and urine cultures, finalized later, were negative (Table 1).

**Table 1:** Laboratory tests on admission APTT: activated partial thromboplastin time; FDP: fibrinogen/fibrin degradation products; PT-INR: prothrombin time–international normalized ratio

Parameters	Value	Reference range
Complete blood count		
White-cell count (per μL)	8,100	4,100-1,5000
Red-cell count (per μL)	4,920,000	4,100,000-5,200,000
Hemoglobin (g/dL)	12.5	11.5-14.4
Platelet count (per μL)	187,000	180,000-580,000
Coagulation tests		
PT-INR	1.1	0.90-1.15
APTT (sec)	27.8	26.9-38.1
FDP (μg/mL)	6.4	<5.0
D-dimer (μg/mL)	2.9	<1.0
Serum chemistry		
Total protein (g/dL)	7.2	6.2-7.7
Albumin (g/dL)	3.5	3.6-4.7
Sodium (mmol/liter)	132	137-144
Potassium (mmol/liter)	4.1	3.6-4.7
Chloride (mmol/liter)	95	101-110
C-reactive protein (mg/dL)	8.29	<0.3
Lactate dehydrogenase (U/liter)	534	175-320
Alanine aminotransferase (U/liter)	34	9-27
Aspartate aminotransferase (U/liter)	39	24-44

The patient was diagnosed with Kawasaki disease, having met five of the major diagnostic criteria: persistent fever, bilateral conjunctival injection, erythema of the lips, bilateral cervical lymphadenopathy, and rash. Treatment was initiated with intravenous immunoglobulin (IVIG) and oral aspirin. Echocardiography showed no evidence of coronary artery abnormalities. Following the initiation of therapy, the patient’s body temperature gradually decreased, returning to normal by the third day of hospitalization. Simultaneously, other clinical symptoms also demonstrated significant improvement.

As described above, the rash, characterized by papules with a tendency to coalesce, prompted consideration of alternative diagnoses, particularly viral infections, at the time of hospitalization. To investigate further, serum antibody titers for various viruses were measured. On the fourth day of hospitalization, the results were obtained. The serum sample collected prior to the administration of IVIG tested positive for EBV viral capsid antigen (VCA)-IgM and VCA-IgG, while the Epstein-Barr nuclear antigen (EBNA) antibody was negative. These findings indicated that the patient was undergoing an acute EBV infection. Additionally, given the coalescing nature of the rash, the possibility of modified measles was also considered, and measles antibody titers were evaluated. The results showed a negative IgM and a positive IgG, effectively ruling out the diagnosis of modified measles.

Retrospectively, the erythematous papules with a tendency to coalesce observed on the patient’s body were consistent with the characteristic rash associated with EBV-related infectious mononucleosis, which can occur following amoxicillin administration. The elevated lactate dehydrogenase levels observed in the blood test were also consistent with this disease. The patient’s symptoms subsequently improved, and she was discharged on the seventh day of illness. At a follow-up outpatient visit after discharge, it was confirmed that there had been no recurrence of symptoms.

## Discussion

The patient initially developed acute otitis media, which resolved with amoxicillin treatment. However, 10 days later, persistent fever ensued, leading to a diagnosis of acute bronchitis and subsequent treatment with clarithromycin. Despite this intervention, the fever persisted, and a generalized rash emerged. Additionally, erythema of the lips and bilateral cervical lymphadenopathy were observed, prompting a clinical diagnosis of Kawasaki disease and the initiation of appropriate treatment. Nevertheless, the rash morphology was not entirely consistent with Kawasaki disease, necessitating further serological evaluation for viral infections. EBV serology revealed elevated antibody titers, indicative of a primary EBV infection. Given the rash characteristics and the temporal association with amoxicillin administration, the final diagnosis was determined to be an amoxicillin-associated rash in EBV-related infectious mononucleosis rather than Kawasaki disease. Although a clarithromycin-induced rash was also considered in the differential diagnosis, the short latency period following drug administration rendered this possibility less likely.

Rashes associated with infectious mononucleosis can occur either in conjunction with amoxicillin administration or independently, without a history of amoxicillin use, as a direct consequence of the infection itself. The former, known as amoxicillin-associated rash, typically presents as papules with a tendency to coalesce approximately 10 days after amoxicillin administration [4,5]. In contrast, rashes occurring solely due to infectious mononucleosis usually appear four to six days after the onset of the disease and are nonspecific, manifesting as various types of rashes. Distinguishing between these two forms of rash can be challenging, as there is no fundamental difference in their presentation. Some studies suggest that the administration of amoxicillin may contribute to the development of rashes in the context of infectious mononucleosis. In the present case, a rash appeared on the 12th day following amoxicillin administration and the second day after the onset of infectious mononucleosis. The rash was characterized by papules with a tendency to coalesce, which is consistent with the presentation of an amoxicillin-associated rash in patients with infectious mononucleosis. Based on both the timing and the characteristics of the rash, it is strongly suspected that amoxicillin administration played a significant role in its development in this case. Incidentally, rashes during EBV-associated infectious mononucleosis have also been reported with antibiotics other than amoxicillin. The frequency of antibiotic-induced rashes in EBV-associated infectious mononucleosis has been reported to vary depending on the class of antibiotics used. According to Chovel-Sella et al. [6], the frequency was highest for amoxicillin (29.5%), followed by cephalosporins (15.4%), macrolides (9.1%), and penicillin (8.6%), with the rate of amoxicillin-induced rashes being significantly higher (p < 0.001). In the present case, the patient had taken clarithromycin the day before the rash appeared. Although the timeline and clinical course make clarithromycin-induced rash unlikely, the possibility cannot be completely ruled out.

Case reports have documented the occurrence of rashes associated with amoxicillin (or ampicillin) administration in the context of infectious mononucleosis [7-20]. In most reported cases, patients were initially misdiagnosed with bacterial tonsillitis or pharyngitis based on findings of the throat and tonsils and were subsequently treated with amoxicillin or ampicillin. These patients developed rashes after beginning antibiotic therapy. Additionally, other cases involved amoxicillin administration for symptoms such as fever and fatigue that were later attributed to infectious mononucleosis, with rashes developing following medication use. In contrast to these scenarios, the present case is notable because amoxicillin was prescribed for otitis media before the onset of symptoms characteristic of infectious mononucleosis. These findings indicate that amoxicillin-associated rashes can occur not only when amoxicillin is administered after the onset of infectious mononucleosis but also when it is taken during the latent phase of EBV infection. This case underscores the importance of obtaining a thorough medical history, including details of medication use during the latent period, when evaluating patients presenting with rashes.

González-Delgado et al. described a separate case involving a seven-year-old girl who developed an amoxicillin rash nine days after initiating amoxicillin treatment for EBV-associated infectious mononucleosis [19]. In that case, diagnostic tests, including prick, intradermal, and patch tests, were conducted with various antibiotics, and only aminopenicillins (amoxicillin and ampicillin) yielded positive results. Historically, prior to the introduction of amoxicillin, ampicillin - another aminopenicillin - was also associated with a high frequency of antibiotic-induced rashes in infectious mononucleosis. Although the precise molecular mechanisms underlying the development of these rashes are not yet fully understood, the similarity in the rash characteristics associated with amoxicillin and ampicillin is thought to result from their shared chemical structure, particularly the presence of an aminobenzyl group. EBV is also known to alter the number and activity of immune cells, such as plasmacytoid dendritic cells, in the blood during its six-week latent period [2]. This phenomenon may be associated with the administration of antibiotics during the latent period, which could have contributed to the appearance of a rash following the onset of infectious mononucleosis. Further research is needed to elucidate the detailed mechanisms by which aminopenicillins contribute to the onset of rashes in the context of EBV-associated infectious mononucleosis.

This case, ultimately diagnosed as an amoxicillin rash associated with infectious mononucleosis, initially met the diagnostic criteria for Kawasaki disease at presentation, prompting the initiation of treatment with IVIG and oral aspirin. In Kawasaki disease, fever typically resolves rapidly following IVIG administration; however, in this case, the resolution of fever was more gradual. The predominant age group for Kawasaki disease is children under five years old, with the highest incidence observed in one- to two-year-olds. However, the patient in this case was seven years old, which falls outside the typical age range for Kawasaki disease. This discrepancy supports the final diagnosis of amoxicillin rash rather than Kawasaki disease in this patient. Coronary artery evaluation was conducted as clinically indicated, and no aneurysms were detected throughout the course of the illness. Amoxicillin rash associated with infectious mononucleosis and Kawasaki disease shares several overlapping clinical features, including fever, rash, and cervical lymphadenopathy. This overlap can complicate the differential diagnosis, particularly during the acute phase. Moreover, since the confirmation of anti-EBV antibody test results typically requires 2-3 days, Kawasaki disease could not be excluded at the time of the initial evaluation. This case underscores the diagnostic challenges posed by overlapping clinical presentations and the importance of maintaining a broad differential diagnosis in such scenarios.

## Conclusions

This case highlights the diagnostic challenges in differentiating amoxicillin-associated rash in EBV-related infectious mononucleosis from Kawasaki disease. Notably, amoxicillin administered for another condition during the latent phase of EBV infection can still trigger the characteristic rash after the onset of infectious mononucleosis. This case underscores the importance of obtaining a detailed medication history, including drugs taken during the latent period, and considering viral etiologies when evaluating febrile illnesses with rashes, particularly when clinical features overlap with systemic inflammatory conditions like Kawasaki disease.
